# Single-Cell RNA Sequencing Analysis of the Heterogeneity in Gene Regulatory Networks in Colorectal Cancer

**DOI:** 10.3389/fcell.2021.765578

**Published:** 2021-11-30

**Authors:** Rui-Qi Wang, Wei Zhao, Hai-Kui Yang, Jia-Mei Dong, Wei-Jie Lin, Fa-Zhong He, Min Cui, Zhi-Ling Zhou

**Affiliations:** Department of Pharmacy, Zhuhai People’s Hospital, Zhuhai Hospital Affiliated with Jinan University, Zhuhai, China

**Keywords:** colorectal cancer, single-cell RNA sequencing, consensus molecular subtypes, gene regulation networks, ERG

## Abstract

Colorectal cancer (CRC) manifests as gastrointestinal tumors with high intratumoral heterogeneity. Recent studies have demonstrated that CRC may consist of tumor cells with different consensus molecular subtypes (CMS). The advancements in single-cell RNA sequencing have facilitated the development of gene regulatory networks to decode key regulators for specific cell types. Herein, we comprehensively analyzed the CMS of CRC patients by using single-cell RNA-sequencing data. CMS for all malignant cells were assigned using CMScaller. Gene set variation analysis showed pathway activity differences consistent with those reported in previous studies. Cell–cell communication analysis confirmed that CMS1 was more closely related to immune cells, and that monocytes and macrophages play dominant roles in the CRC tumor microenvironment. On the basis of the constructed gene regulation networks (GRNs) for each subtype, we identified that the critical transcription factor *ERG* is universally activated and upregulated in all CMS in comparison with normal cells, and that it performed diverse roles by regulating the expression of different downstream genes. In summary, molecular subtyping of single-cell RNA-sequencing data for colorectal cancer could elucidate the heterogeneity in gene regulatory networks and identify critical regulators of CRC.

## Introduction

Colorectal cancer (CRC) is a widespread cancer that accounts for almost 10% of all cancer-related deaths (Sung et al., 2021). CRC is characterized by gastrointestinal tumors with high intratumoral heterogeneity (Budinska et al., 2013), and previous studies have identified important clinical subtypes using the accumulated gene-expression profile data (Schlicker et al., 2012; De Sousa et al., 2013; Marisa et al., 2013; Sadanandam et al., 2013). For instance, the mesenchymal-like subgroup shows a high degree of stromal infiltration and a poor response to standard chemotherapy (De Sousa et al., 2013; Sadanandam et al., 2013; Song et al., 2016; Trinh et al., 2017), resulting in a poor prognosis (Isella et al., 2015). On the basis of gene-expression analysis of nearly 4,000 primary tumors, Guinney et al. (2015) classified CRC into four consensus molecular subgroups (CMS1 to CMS4), and these categorizations have received extensive attention. CMS1 shows high immune infiltration and activation levels, along with microsatellite instability (MSI-H). CMS2 is defined by WNT and MYC pathway activation with poor intratumoral immune-cell infiltration. CMS3 is associated with KRAS mutations and reflects metabolic dysregulation with higher activity in glutaminolysis and lipidogenesis (Stintzing et al., 2016). CMS4 presents with the highest stromal infiltration and activation of epithelial–mesenchymal transition (EMT), making it resistant to chemotherapy. Recent studies have demonstrated that these tumors are highly heterogeneous, and that a single patient sample may consist of cells with multiple subtypes (Yeo and Guan, 2017). Thus, the intratumoral heterogeneity of CRC should be investigated to facilitate precision medicine.

Single-cell RNA sequencing (scRNA-seq) can help quantify the transcriptome status of tumor tissue at a single-cell resolution, facilitating evaluations of genetic heterogeneity. For example, Karaayvaz et al. (2018) adopted scRNA-seq to reveal a distinct subpopulation of epithelial cells in the tumor microenvironment (TME) that could be associated with the long-term survival of triple-negative breast cancer (TNBC) patients. Zhang et al. (2020) performed scRNA-seq analyses on the stromal and immune populations from patients with CRC and identified specific conventional dendritic cells (cDCs) and macrophage subsets as key mediators of cellular crosstalk in the tumor microenvironment. scRNA-seq analysis has also ushered in considerable development. Using scRNA-seq data, Iacono et al. (2019) constructed gene regulation networks (GRNs) with a global regulatory model, which could be helpful for elucidating the heterogeneity of gene regulatory networks and identifying critical regulators.

In this study, we comprehensively analyzed the CMS of CRC patients by using single-cell RNA-seq data. The malignant cells were identified by inferred copy number variation (CNV), and the CMS were assigned using CMScaller for all malignant cells. Gene set variation analysis (GSVA) showed consistent pathway activity differences among subtypes in previous studies. Cell–cell communication analysis based on ligand–receptor interactions confirmed that the CMS1 was more closely related to immune cells, and monocytes and macrophages play dominant roles in the CRC tumor microenvironment. On the basis of the constructed GRNs for each subtype, we identified that the critical transcription factor (TF) ERG was universally activated and upregulated in all CMS in comparison with normal cells. The dysregulation of *ERG* exerts diverse effects by regulating the expression of different downstream genes, which could be associated with the gene regulatory network heterogeneity and tumor progression of CRC. Further analysis of The Cancer Genome Atlas (TCGA) dataset confirmed the worse prognostic phenotype of CMS4 and the immune infiltration status of CMS1 and revealed the high heterogeneity of the bulk tumor sample.

## Materials and Methods

### Data Collection and Processing

We downloaded the scRNA-seq data of nine CRC patients (P0825, P1212, P1228, P0104, P0305, P0411, P0413, P0720, and P0728) from the GEO dataset (https://www.ncbi.nlm.nih.gov/geo/) with the accession ID GSE146771 (Zhang et al., 2020). The data were then normalized according to the flowchart mentioned by Sun et al. (2021) The gene-expression profiles and clinical information of TCGA CRC patients were downloaded from the UCSC Xena Browser (https://xenabrowser.net/datapages/). The proteome data of the CRC patients were downloaded from the CPTAC dataset (https://cptac-data-portal.georgetown.edu/).

### Identification of Malignant Epithelial Cells and CMS

We inferred the CNVs for 1,123 epithelial cells from the scRNA-seq dataset by using the “infercnv v1.6.0” R package (https://github.com/broadinstitute/infercnv). Cells derived from normal samples were used as a control reference for CNV inference. After inferring the CNVs for all cells, the cells were clustered into two subgroups, and the cluster with the higher CNV standard deviation was renamed as malignant cells. Then, we classified the malignant cells into four subtypes based on their expression profiles by using the R package “CMScaller v2.0.1.” (Eide et al., 2017) The cells that could not be assigned to any subgroup were removed.

### Cell–Cell Communication Analysis

The immune and stromal-cell annotations were curated from a previous study (Zhang et al., 2020). To investigate the communications among all cell types, including the four subtypes of tumor cells, immune cells, and stromal cells, we applied the *Python* package “CellPhoneDB” (Efremova et al., 2020) to estimate the potential ligand–receptor pairs. The pairs with *p* < 0.05 were considered to show significant interactions between 2 cell types and were evaluated for further analysis.

### GSVA Analysis and Pathway Enrichment Analysis

The cancer hallmark and KEGG pathways were downloaded from the MSigDB (Liberzon et al., 2015). GSVA analysis for each cell was performed using the R package “GSVA v1.38.2.” (Hänzelmann et al., 2013) Pathway enrichment analysis of a specific set of genes was performed by using the R package “clusterProfiler v3.18.1.” (Yu et al., 2012).

### Construction of GRNs

We constructed subtype-specific GRNs for normal endothelial cells and the four CMS. First, we identified the significantly co-expressed TF-target pairs based on gene-expression profiles, and then, we removed genes that were not enriched in the binding motifs of the corresponding TF for each TF-target pair. To minimize the false-discovery rate, we used only the remaining TF-target pairs with an ES > 1. The co-expression analysis and motif-enrichment analysis were performed using the *Python* package “pySCENIE.” (Aibar et al., 2017) The GRN network was visualized by “Cytoscape” software (Shannon et al., 2003).

### Critical Regulator Identification

To identify the critical regulators that play important roles in the GRN of each subtype, we adopted five measurements to evaluate the centrality of each node, as mentioned previously (Iacono et al., 2019). These measurements were PageRank, degree, eigenvalue, betweenness, and closeness. Then, we transferred the scores of each measurement into rank levels. The final score of each node was defined as the sum of all five sorted indicators. A lower score indicated higher centrality, which represented the importance of a selected node.

### Survival Analysis

The log-rank test that compares the survival differences of two groups at each observed event time was performed by the R “survival v3.2-11” package (https://cran.r-project.org/web/packages/survival). Kaplan-Meier analysis was applied to obtain a survival curve plot of CRC subtypes.

### Immune-Cell Abundance and Tumor Purity

CIBERSORT (Chen et al., 2018) was used to retrieve the immune-cell components in CRC samples on the basis of the gene-expression profiles of TCGA samples. The immune infiltration, stromal infiltration, and tumor purity were evaluated using the R package “estimate v1.0.13.” (Yoshihara et al., 2013).

## Results

### CMS of CRC Patients

**FIGURE 1:**
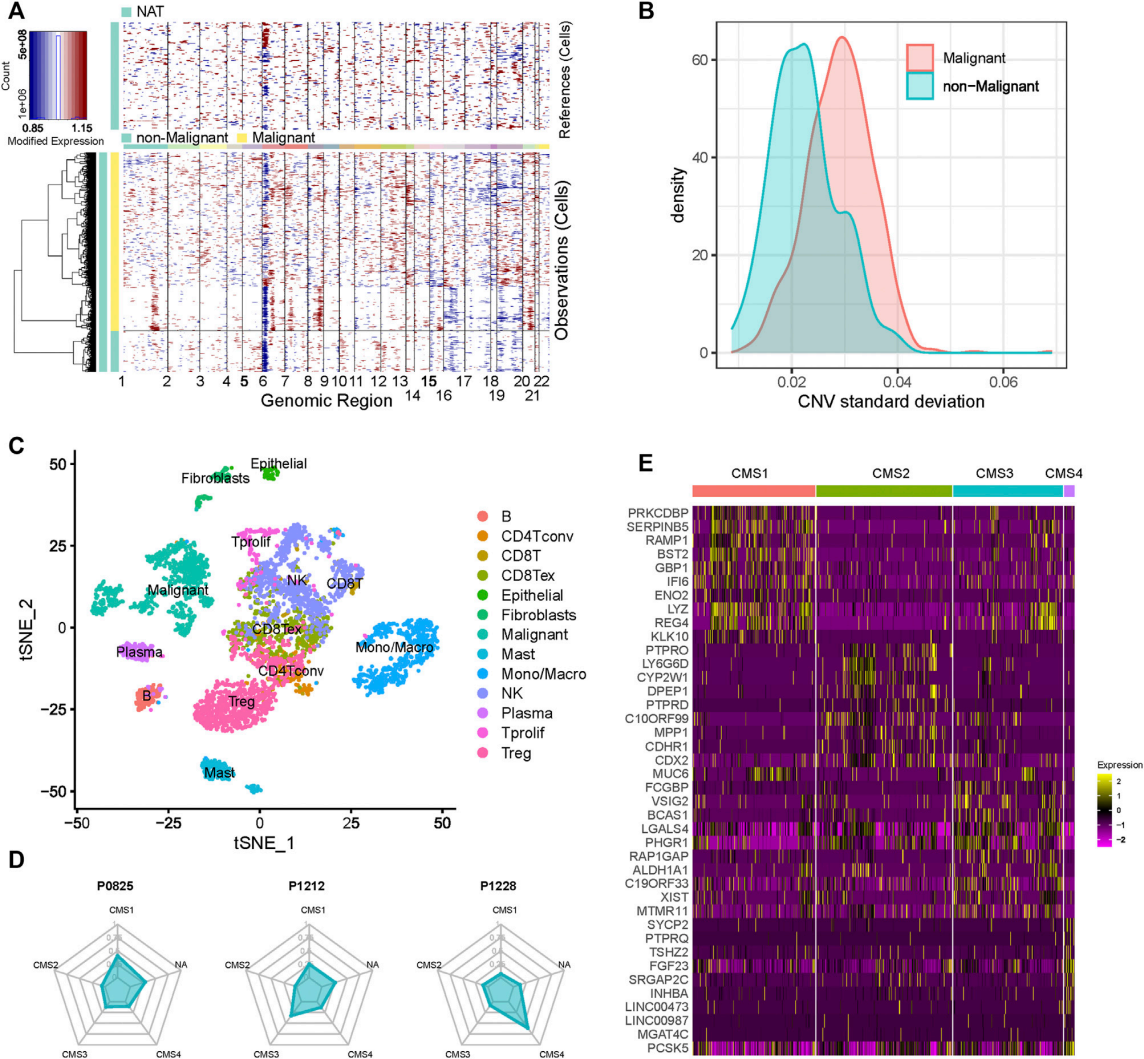
Cell composition of CRC patients **(A)** Heatmap of the inferred CNVs across 1,123 epithelial cells **(B)** Standard deviation of the CNVs of the cells in each cluster **(C)** t-SNE plot of the scRNA-seq data **(D)** CMS composition of each CRC patient **(E)** Heatmap of the markers’ expression in each subgroup.

Using the gene-expression profile data and metadata from the scRNA-seq dataset, we inferred the CNVs for 1,123 epithelial cells. These epithelial cells were then classified into 913 malignant and 211 non-malignant cells. As shown in Figures 1A,B, the malignant cells displayed a relatively higher standard deviation of the CNV than the non-malignant cells. Next, we applied t-Distributed Stochastic Neighbor Embedding (t-SNE) to perform dimension reduction for all cells derived from these patients, and the immune-cell and stromal-cell annotation were curated from a previous study (Zhang et al., 2020). Malignant cells showed distinct boundaries with the other cells (Figure 1C). As expected, B-cells highly expressed markers such as MS4A1 and CD79A; T cells exhibited significant upregulation of markers such as CD3E, CD4, CD3G and CD8A; CD33, and CD14 were significantly elevated in monocytes and macrophages (Supplementary Figure S1).

We then assigned CMS for all malignant cells by using CMScaller. Malignant cells were classified into four well-known molecular subtypes: CMS1, CMS2, CMS3, and CMS4 (Supplementary Table S1). A total of 659 cells were assigned to the different subgroups, and 254 cells did not belong to any subgroup. We found that genes that showed significantly different expression levels in different subtypes had a certain degree of commonality, but many genes also belonged to distinct subtypes (Figure 1D and Supplementary Figure S2). After mapping the cells to the patients, we found that almost none of the patients contained only one subtype of cells (Figure 1E and Supplementary Figure S2). As shown in the figure, P0825 and P0413 exhibited relatively higher numbers of CMS1 cells, while P0411 and P0728 showed more CMS2 cells. These results confirm that CRC is a highly heterogeneous tumor, and that a patient sample may consist of cells with multiple subtypes.

**FIGURE 2:**
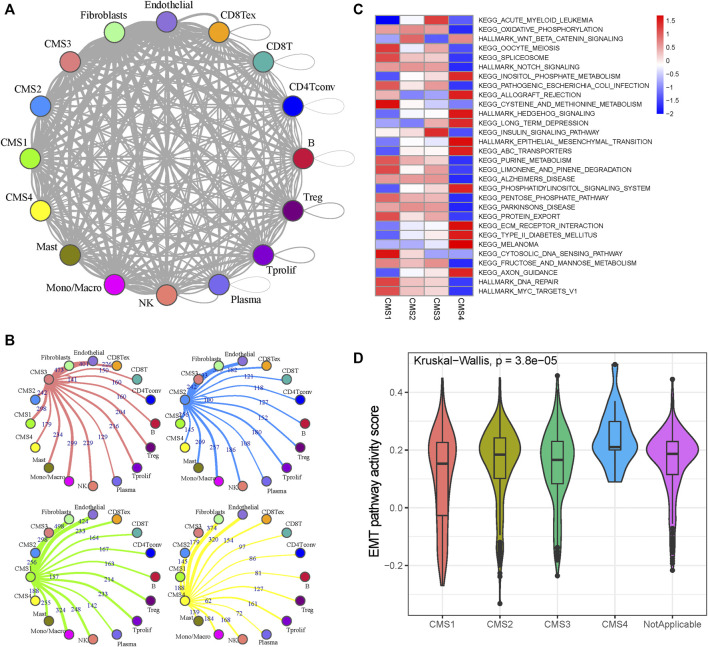
Cell-cell interaction and pathway activity analysis **(A)** Cell-cell interaction network of different cell types. The node size represents the number of interactions. The width of the edge represents the number of significant ligand-receptor interactions in the 2 cell types **(B)** Cell-cell interaction network of CMS and other cells **(C)** Differences in the enrichment of the pathways across the five molecular subtypes **(D)** Violin plots of GSVA enrichment scores of the EMT pathway of the four molecular subtypes.

The *Python* CellPhoneDB package was used to investigate the cell–cell crosstalk in the tumor microenvironment of CRC (Figure 2A). The number of ligand–receptor pairs presented in Figure 2B and Supplementary Figure S3 suggests that monocytes and macrophages play dominant roles in the CRC tumor microenvironment (Supplementary Table S3). The CMS1 subtypes are more closely related to immune cells than the other subtypes (Chi-square test *p*-value, CMS1:CMS2 = 1.40e-20; CMS1:CMS3 = 1.54e-13; CMS1:CMS4 = 7.38e-25), consistent with the definition that CMS1 shows high immune infiltration and activation levels. To reveal the cells’ activity in the hallmark and Kyoto Encyclopedia of Genes and Genomes (KEGG) gene sets, we performed GSVA analysis for each cell to evaluate the pathway activities (Supplementary Table S4). As shown in Figure 2C, CMS1 was defined by microsatellite instability (MSI-H) status, and CMS1 subgroup cells showed significantly higher DNA repair pathway activity. CMS2 was defined by WNT and MYC pathway activation with poor intratumoral immune-cell infiltration, and the WNT signaling activity was elevated in the CMS2 subgroup. CMS4 showed the highest stromal infiltration, and the activation of the EMT pathway made it resistant to chemotherapy, with the EMT pathway activity score being significantly higher than those in the other subgroups (Figure 2D).

### Construction of GRNs for Each Subgroup

**FIGURE 3:**
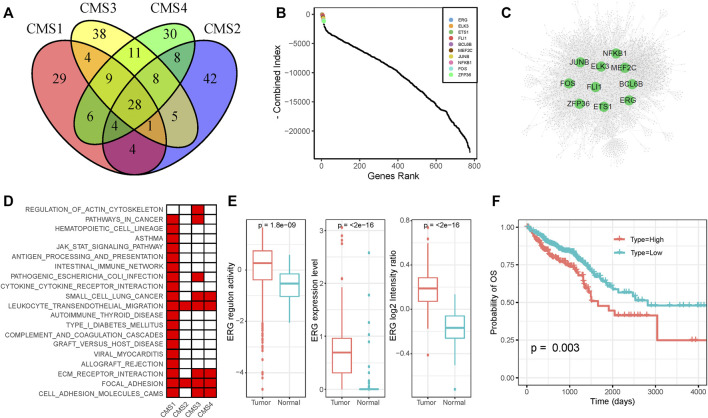
Functional diversity of ERG **(A)** Venn plot of functional TFs of the four CMS **(B)** The importance of TFs among all four subtypes **(C)** The gene regulation network of CRC samples **(D)** KEGG annotation of ERG-regulated genes in each subtype **(E)** Expression level and activity of ERG in normal samples and tumor samples **(F)** Survival analysis of CRC patients on the basis of ERG expression. Lower expression of ERG was associated with a better clinical outcome.

The TF-target regulation network could help researchers clarify potential dysfunctional regulators in cancer. To identify the key regulators that play critical roles in CRC, we constructed subtype-specific GRNs for normal epithelial cells and four CMS. We identified the significantly co-expressed TF-target pairs based on gene-expression profiles and removed genes that were not enriched in the binding motifs of the corresponding TF for each TF-target pair. To minimize the false-discovery rate, we only used the remaining TF-target pairs with an enrichment score (ES) of >1 (Supplementary Table S5). The occurrences of TFs and the constructed GRNs for each subtype are shown in Supplementary Figures S4,5. We found that different subtypes share some of the same target genes and TF-target pairs; they also have their own specific regulatory relationships (Supplementary Figure S6). The key regulators of each subtype are also specific and shared. As shown in Figure 3A, the results identified 29, 38, 30, and 42 specific regulators for the CMS1, CMS2, CMS3, and CMS4 subgroups, respectively, and 28 regulators were shared by all subtypes. To define the key regulators in CRC, we adopted five methods to calculate the importance of genes in the GRNs for each subtype and normal cells (Supplementary Table S6). The combined value and GRNs of the four subtypes are displayed in Figures 3B,C. Many well-known regulators are ranked in the top 10. ELK3 elevates the expression of HIF-1*α* and promotes the migration of liver cancer stem cells (Lee et al., 2017). The constitutive NF-*κ*B signaling pathway has already been proven to serve as a regulator of the immune response in several cancers (Horst et al., 2009; Sakamoto et al., 2009).

As shown in the figure, *ERG* was ranked the highest among the four subtypes, and it was not dominant in normal cells, indicating its potential oncogenic function in CRC. *ERG* shows nuclear and cytoplasmic expression in several tissues, which has been identified as a key factor for prostate cancer (Adamo and Ladomery, 2016). We found that *ERG* regulated diverse genes in different subtypes. KEGG pathway enrichment analysis revealed that the targets of *ERG* in different subtypes participated in diverse roles (Figure 3D). Based on the scRNA-seq dataset, we found that the expression level and regulon activity were significantly downregulated in normal cells (Figure 3E). Furthermore, we evaluated the expression of the *ERG* gene based on the CRC proteome profile in the Clinical Proteomic Tumor Analysis Consortium (CPTAC) and found a significantly higher expression level in tumor samples than in normal samples (Figure 3E). On the basis of the clinical information and gene-expression data in the TCGA dataset, we performed survival analysis to show that higher expression of *ERG* was associated with a poorer prognosis (Figure 3F). In conclusion, the dysregulation of *ERG* influences diverse targets in different CRC subtypes, which may be responsible for the intratumoral heterogeneity in CRC. Moreover, *ERG* gene expression was negatively correlated with patient outcomes, indicating that *ERG* might be a potential drug target for CRC.

### The CMS Index Functions as a Biomarker of CRC

**FIGURE 4:**
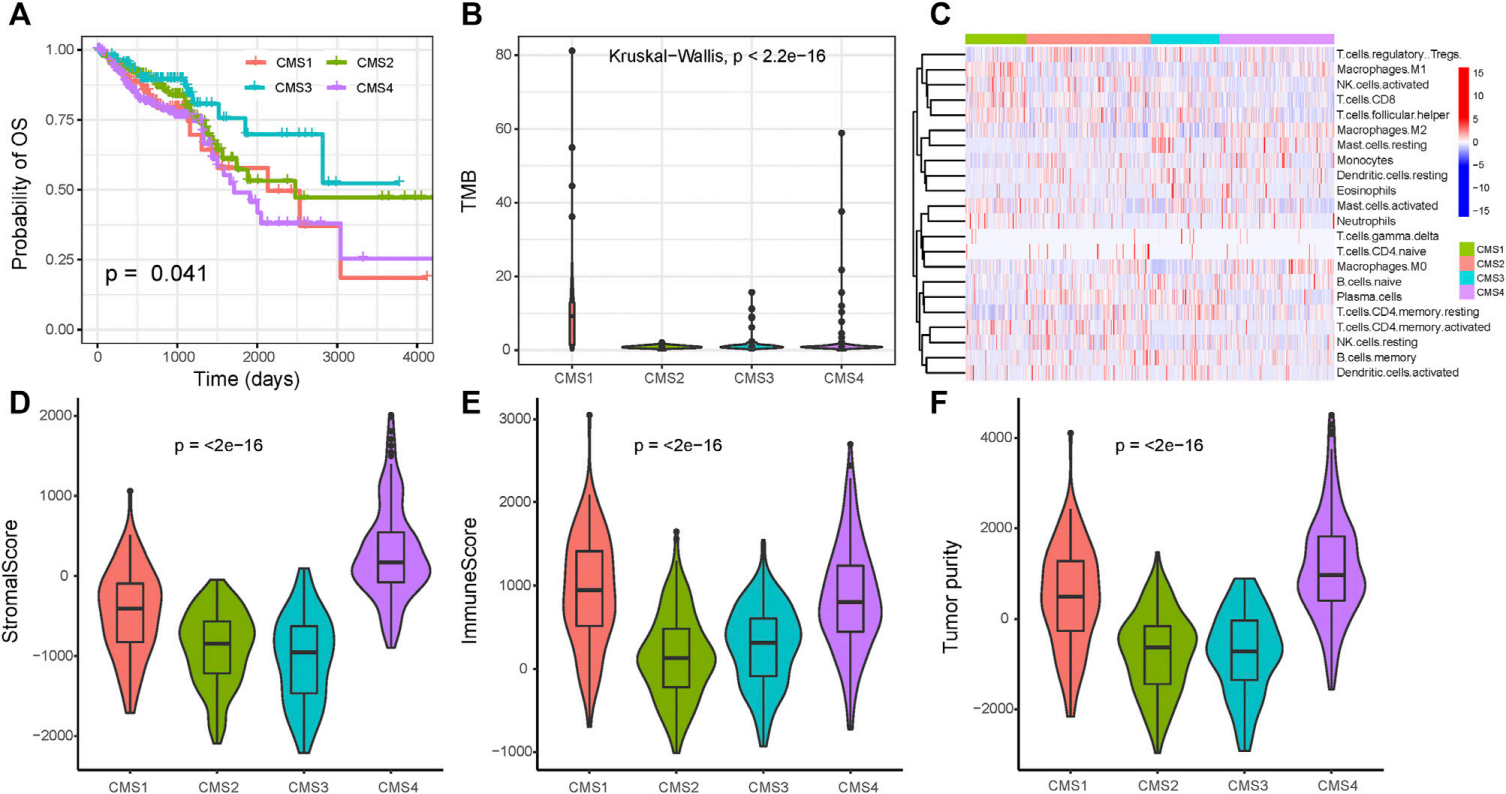
Immune status among the four subgroups **(A)** Overall survival for the four CMS **(B)** Tumor mutation burden among the three subtypes **(C)** Immune-cell infiltration among the three subtypes **(D–F)** The immune microenvironment among the three groups.

**FIGURE 5:**
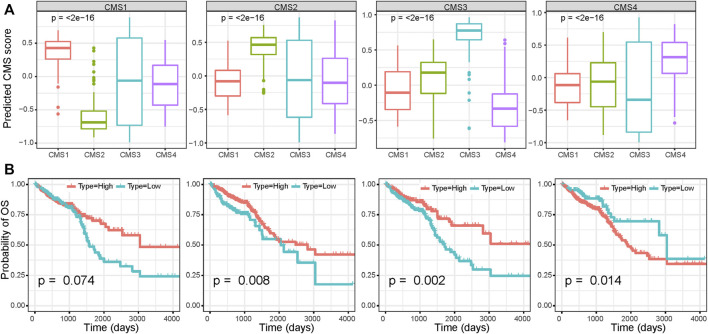
Survival analysis based on the CMS index **(A)** CMS index of each CMS subgroup **(B)** Survival analysis of CRC patients based on the CMS index.

Using the markers of each subtype calculated by the scRNA-seq data, we performed GSVA analysis to determine the relative abundance of each of the CMS in TCGA samples, which we named as CMS index. On the basis of the relative CMS scores, we classified the TCGA samples into CMS1, CMS2, CMS3, and CMS4 subgroups (Supplementary Table S7); the CMS4 subgroup had the worst overall survival, as shown in Figure 4A, which is consistent with the findings of previous studies. CMS1 was defined by microsatellite instability (MSI-H) status, and we found a significantly higher tumor mutation burden (TMB) in the CMS1 subgroup (Figure 4B). We then estimated the relative immune-cell abundance of TCGA samples by using the CIBERSORT software. The CMS1 subgroup displayed significantly higher infiltration of CD8^+^ T cells, natural killer cells, and M1 macrophages, while CMS4 displayed a higher level of M2 macrophages (Figure 4C and Supplementary Figure S7). Previous studies have shown that CMS1 is immune-activated and CMS4 is immune-inflamed with the expression of multiple immune checkpoint inhibitors, while CMS2 shows an immune desert status and CMS3 excluded immune cells. On the basis of the ESTIMATE algorithm, we quantified the scores for stromal infiltration and immune infiltration (Figures 4D–F). CMS4 exhibited a higher level of stromal-cell infiltration, whereas CMS1 showed a high level of immune-cell infiltration. We further evaluated the functional role of the CMS index in CRC prognosis. As shown in Figure 5, the CMS scores were significantly higher in the corresponding identified CMS, and a higher CMS4 score was associated with poorer clinical outcomes. These results further confirmed the poor prognostic phenotype of CMS4 and the immune infiltration status of CMS1, and elucidated the high heterogeneity of the bulk tumor sample.

## Discussion

CRC is a gastrointestinal tumor with high intratumoral heterogeneity (Budinska et al., 2013). Previous studies have identified important clinical subtypes based on accumulated data from gene-expression profiles (Schlicker et al., 2012; De Sousa et al., 2013; Marisa et al., 2013; Sadanandam et al., 2013). In this study, we comprehensively analyzed the CMS of CRC patients by using single-cell RNA-sequencing data. Malignant cells were extracted based on CNV status and then assigned to different CMS by using CMScaller. The fact that almost none of the patients showed only one subtype of cells indicated that CRC is a highly heterogeneous tumor, and a patient sample may consist of cells with multiple subtypes. GSVA analysis showed consistent pathway activity differences among the subtypes in previous studies. In the present study, cell–cell communication analysis based on ligand–receptor interactions confirmed that CMS1 subtypes are more closely related to immune cells, and that monocytes and macrophages play dominant roles in the CRC tumor microenvironment. On the basis of the constructed GRNs of each subtype, we identified that the critical TF *ERG* was universally activated and upregulated in all CMS in comparison with normal cells. The dysregulation of ERG performs diverse roles by regulating the expression of different downstream genes, which could be associated with the heterogeneity of the gene regulatory networks and the progression of CRC. Further analysis of the TCGA dataset confirmed the poor prognostic phenotype of CMS4 and the immune infiltration status of CMS1 and revealed the high heterogeneity of the bulk tumor sample.

Tumor heterogeneity has been the subject of many recent studies and remains a topic of interest in cancer-related research (McGranahan and Swanton, 2015; Andor et al., 2016). Although the different mutation statuses in CRC samples have been evaluated in some studies (Kim et al., 2015; Sottoriva et al., 2015), research on molecular heterogeneity within tumors remains limited. Previous studies have revealed four CMS of CRC (Guinney et al., 2015); however, the key regulators of these subtypes remain unresolved. In this study, we constructed subtype-specific GRNs to identify reliable key regulators. The findings revealed that the *ERG* gene was ranked the highest among the four subtypes, and it was dismissed in normal cells, indicating its potential oncogenic function in CRC. *ERG* shows nuclear and cytoplasmic expression in several tissues, and has been identified as a key factor in prostate cancer (Adamo and Ladomery, 2016). KEGG pathway enrichment analysis revealed that the targets of *ERG* in different subtypes participated in diverse roles. The dysregulation of ERG influences diverse targets in different CRC subtypes, which may be responsible for the intratumoral heterogeneity in CRC. Moreover, *ERG* gene expression was negatively correlated with patient outcome, indicating that *ERG* might be a potential drug target for CRC.

Revealing molecular abnormalities and the regulatory mechanisms in tumors based on single-cell sequencing data analysis is a major trend in future research (Stuart and Satija, 2019), and is something we have been working towards. We have made some findings based on existing data and identified a potential biomarker ERG gene, our article still has some limitations. Although ERG gene was supported as a key factor by experimental data in other cancer types, more robust experimental validation is needed in CRC. We will further validate the potential of ERG genes as drug targets in CRC based on comprehensive experiments. There is also some correlation between CMS subtypes and clinical information as previous reported (Guinney et al., 2015), but due to the limitations of single-cell data, the number of patients is too small to correlate CMS subtypes and clinical features well, and judgments from individual patients alone are prone to bias. We will continue to collect newly published data on single-cell sequencing of CRC and integrate different datasets for a more comprehensive analysis.

## Data Availability

Publicly available datasets were analyzed in this study. This data can be found here: Publicly available datasets were analyzed in this study. These data can be found at: The Cancer Genome Atlas (TCGA), Datasets link: https://xenabrowser.net/datapages/. And Gene Expression Omnibus (GEO), Datasets link: https://www.ncbi.nlm.nih.gov/gds/.

## Supplementary Material

Supplementary Figure S1 Immune cell markers expression of the scRNA-seq dataset.

Supplementary Figure S2 CMS subtype composition of each CRC patient.

Supplementary Figure S3 Cell-cell interaction network of the immune cells and stromal cells.

Supplementary Figure S4 The word cloud map indicating the degree of TFs in normal cells and four CRC subtype-specific GRNs.

Supplementary Figure S5 GRNs of normal cells and four molecular subtypes.

Supplementary Figure S6
**(A)** Venn diagram of targets of the four molecular subtype-specific GRNs. **(B)** Venn diagram of TF-target pairs of the four molecular subtype-specific GRNs.

Supplementary Figure S7 Immune cells the among four subgroups.

Supplementary Table S1 CMS subtype of scRNA dataset.

Supplementary Table S2 Top expressed markers of different subtypes.

Supplementary Table S3 The ligand-receptor pairs s in the CRC tumor microenvironment.

Supplementary Table S4 KEGG and Hallmark pathways enrichment scores of different subtypes.

Supplementary Table S5 Number of candidate TF-target regulations in five GRNs.

Supplementary Table S6 Key regulators of normal cells and four molecular subtypes.

Supplementary Table S7 CMS subtype and ESTIMATE scores of TCGA samples.

## Abbreviations

cDC, conventional dendritic cell; CNV, copy number variation; CPTAC, Clinical Proteomic Tumor Analysis Consortium; CRC, colorectal cancer; EMT, epithelial–mesenchymal transition; ES, enrichment score; GRNs, gene regulation networks; GSVA, Gene set variation analysis; KEGG, Kyoto Encyclopedia of Genes and Genomes; MSI-H, microsatellite instability; scRNA-seq, single-cell RNA sequencing; t-SNE, t-distributed stochastic neighbor embedding; TCGA, The Cancer Genome Atlas; TF, transcription factor; TMB, tumor mutation burden; TME, tumor microenvironment; TNBC, triple-negative breast cancer.
