# Supplementary figures and images for: Single-Cell RNA Sequencing Analysis of the Heterogeneity in Gene Regulatory Networks in Colorectal Cancer
Source: Front Cell Dev Biol. 2021 Nov 30;9:765578. doi: 10.3389/fcell.2021.765578 (PMC8669944; doi:10.3389/fcell.2021.765578)

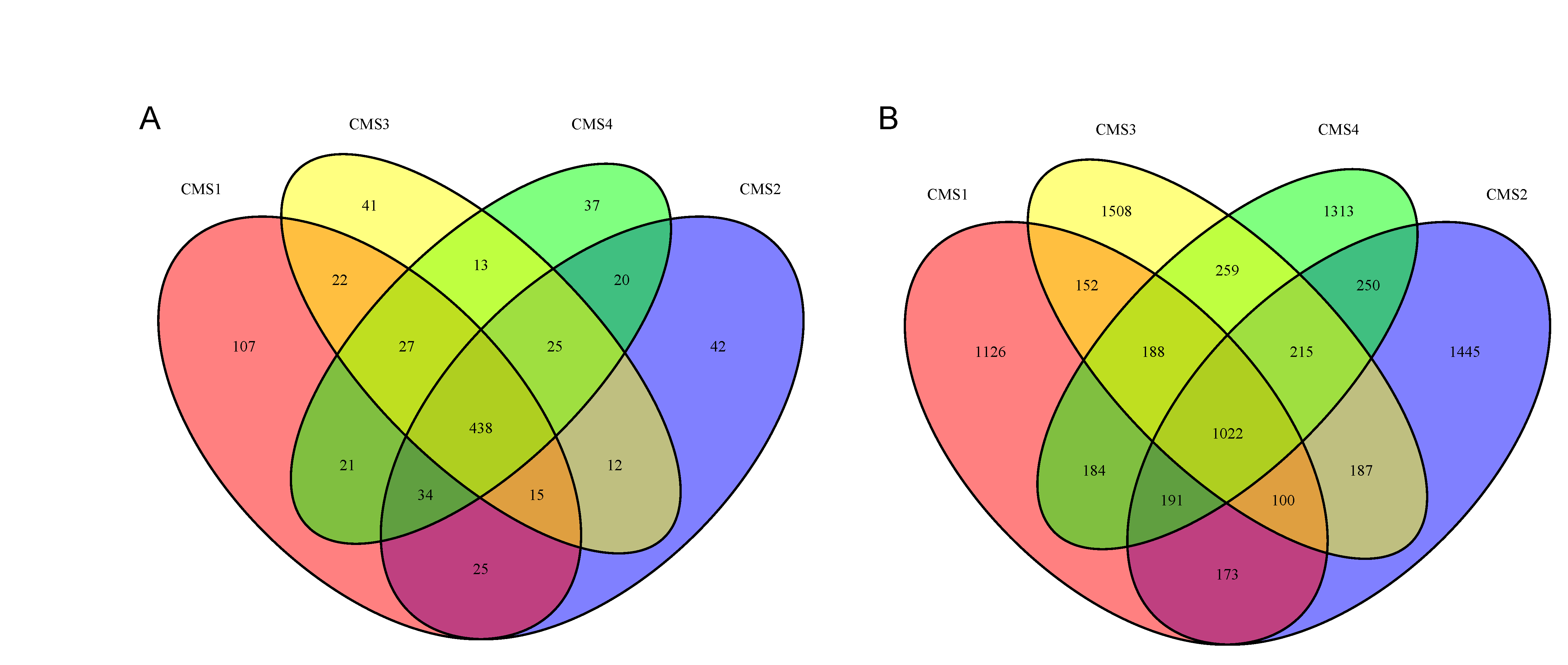

Supplement: Supplementary file 3 [file Image6.TIF]

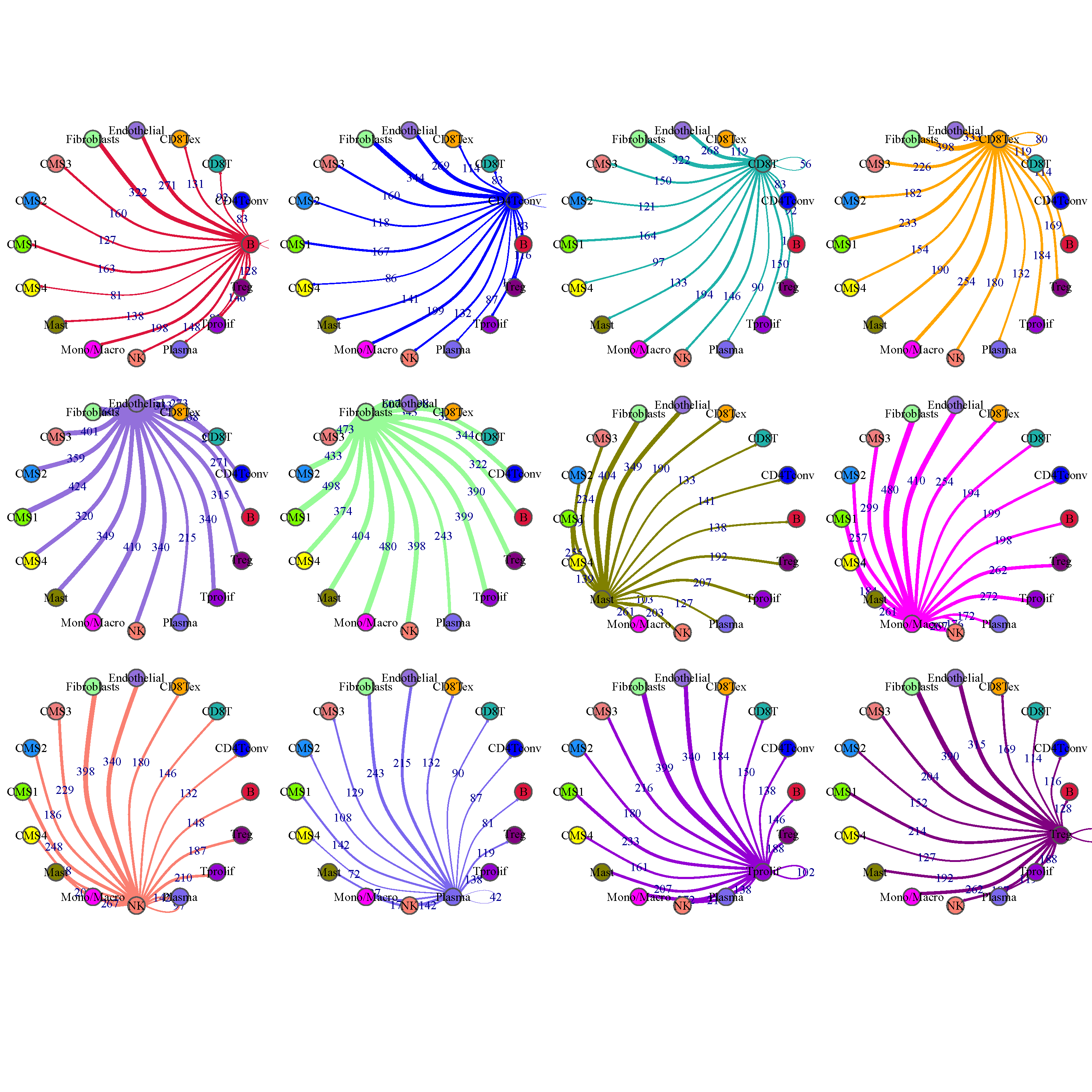

Supplement: Supplementary file 4 [file Image3.TIF]

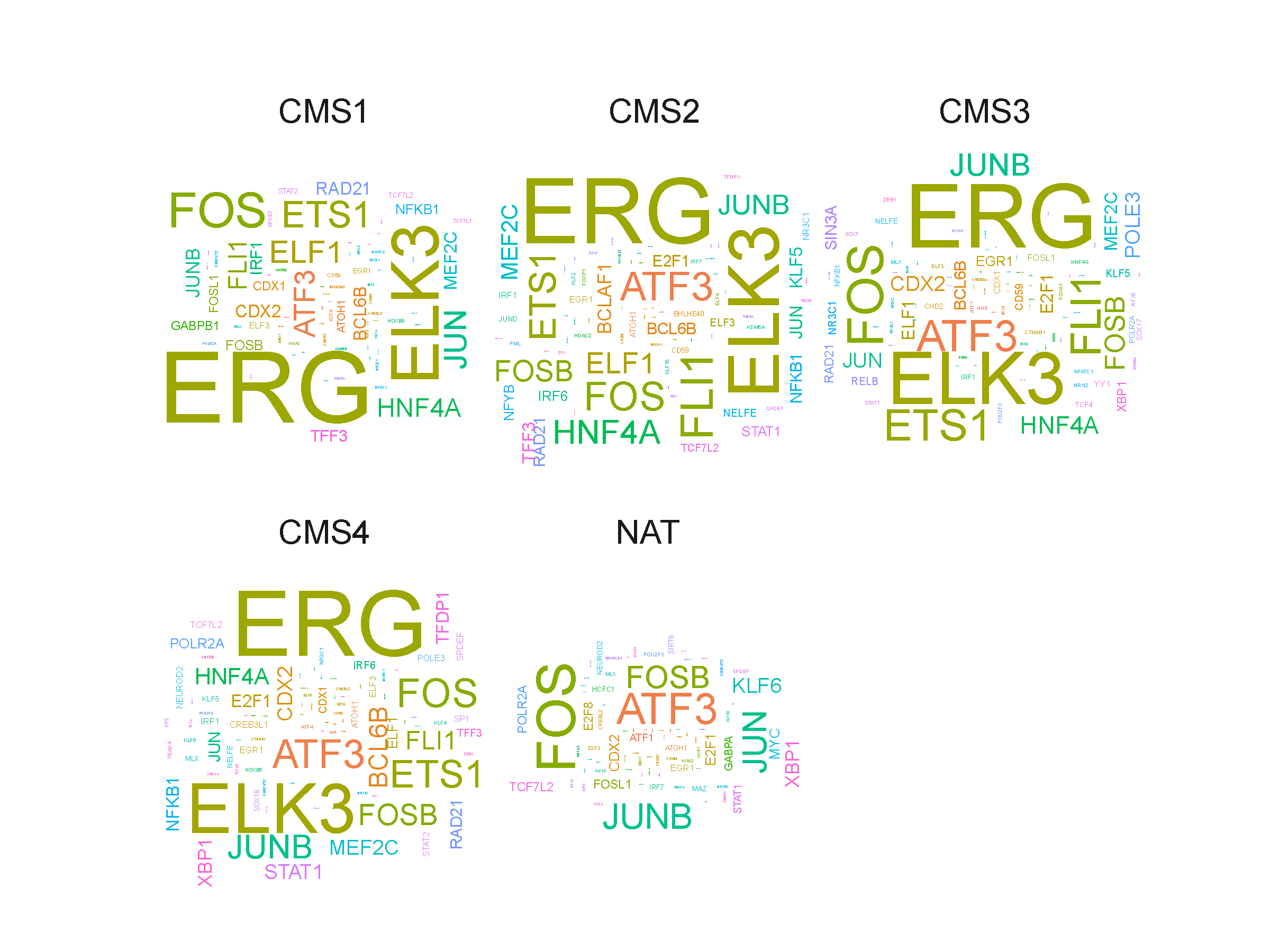

Supplement: Supplementary file 5 [file Image4.TIF]

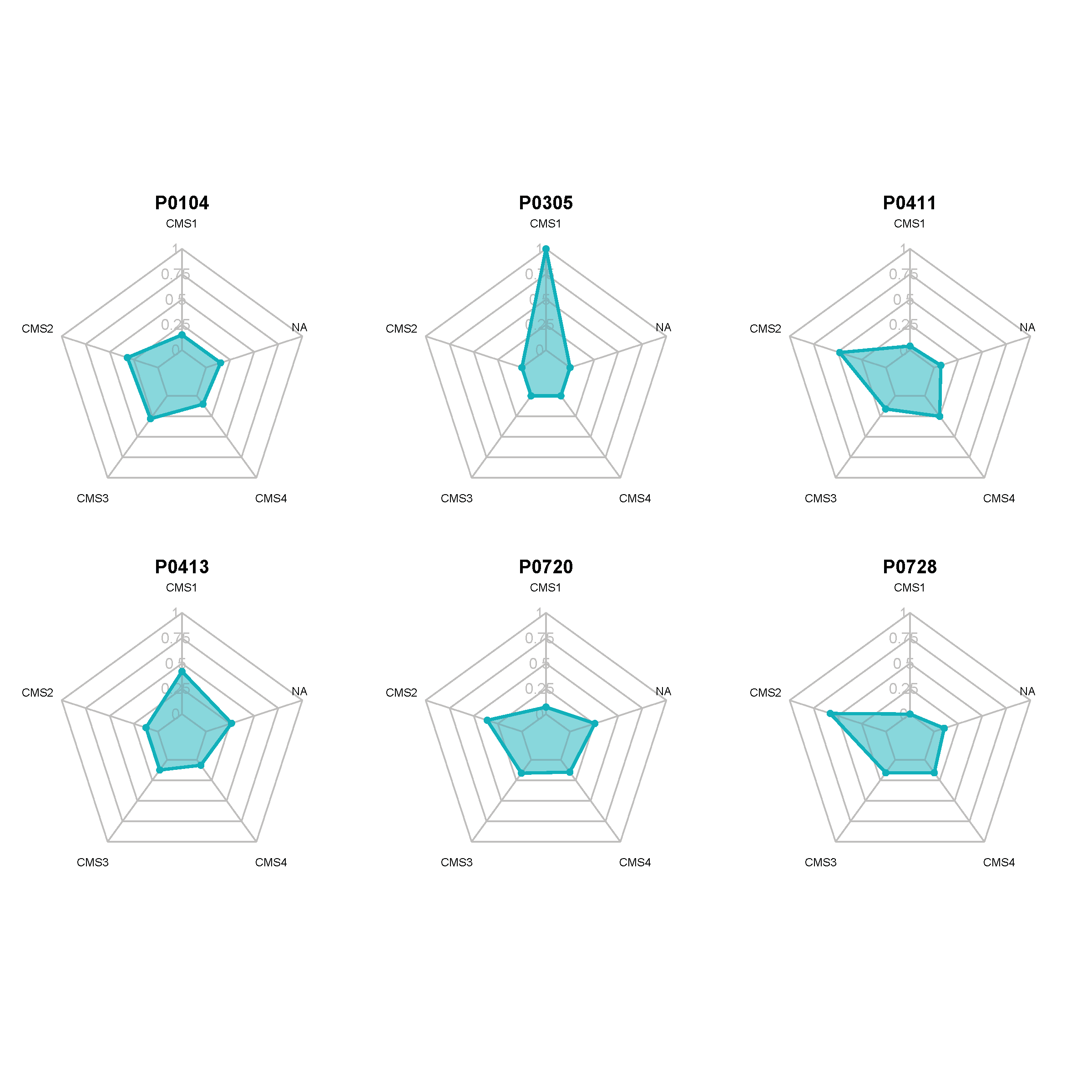

Supplement: Supplementary file 6 [file Image2.TIF]

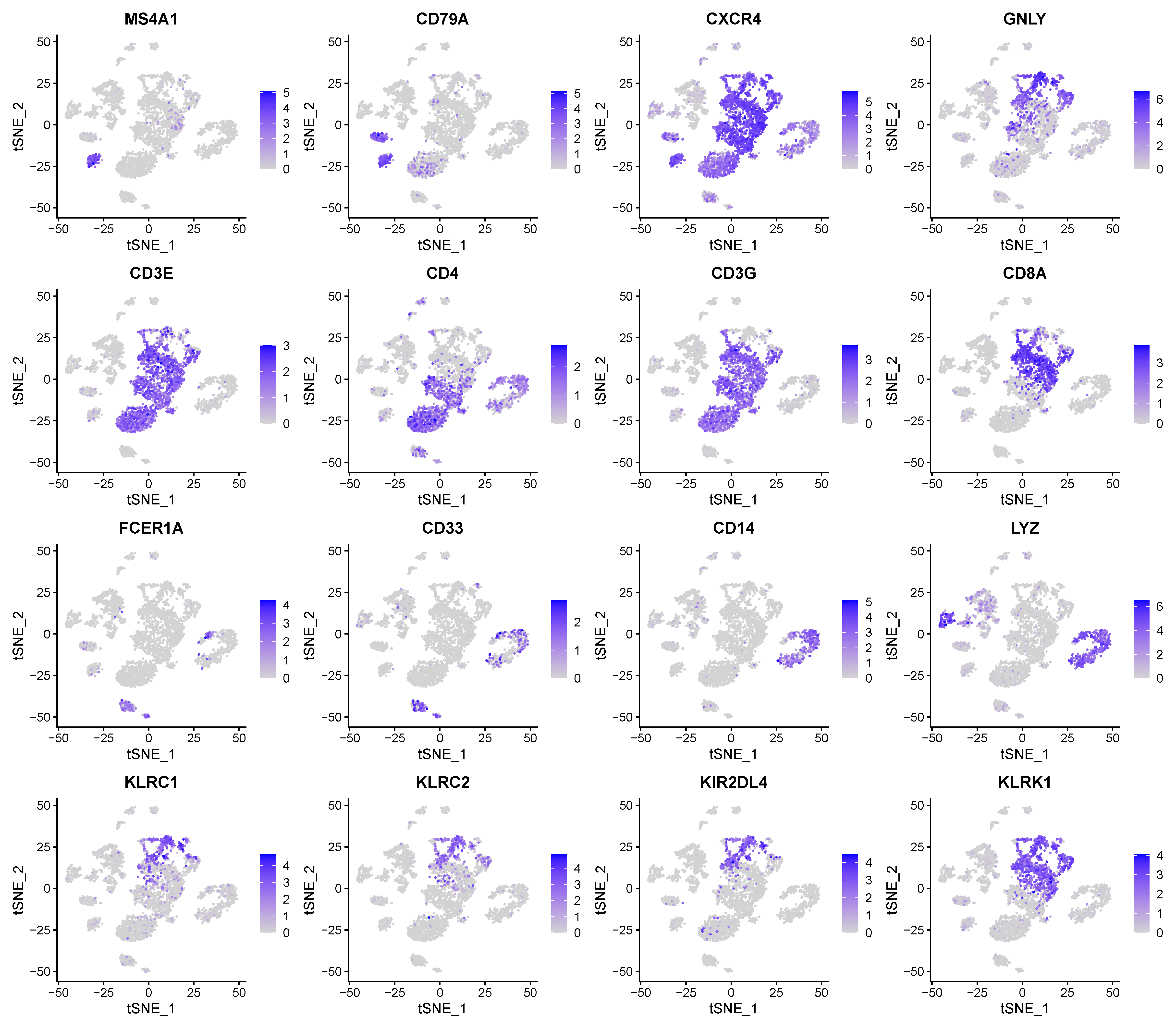

Supplement: Supplementary file 7 [file Image1.TIF]

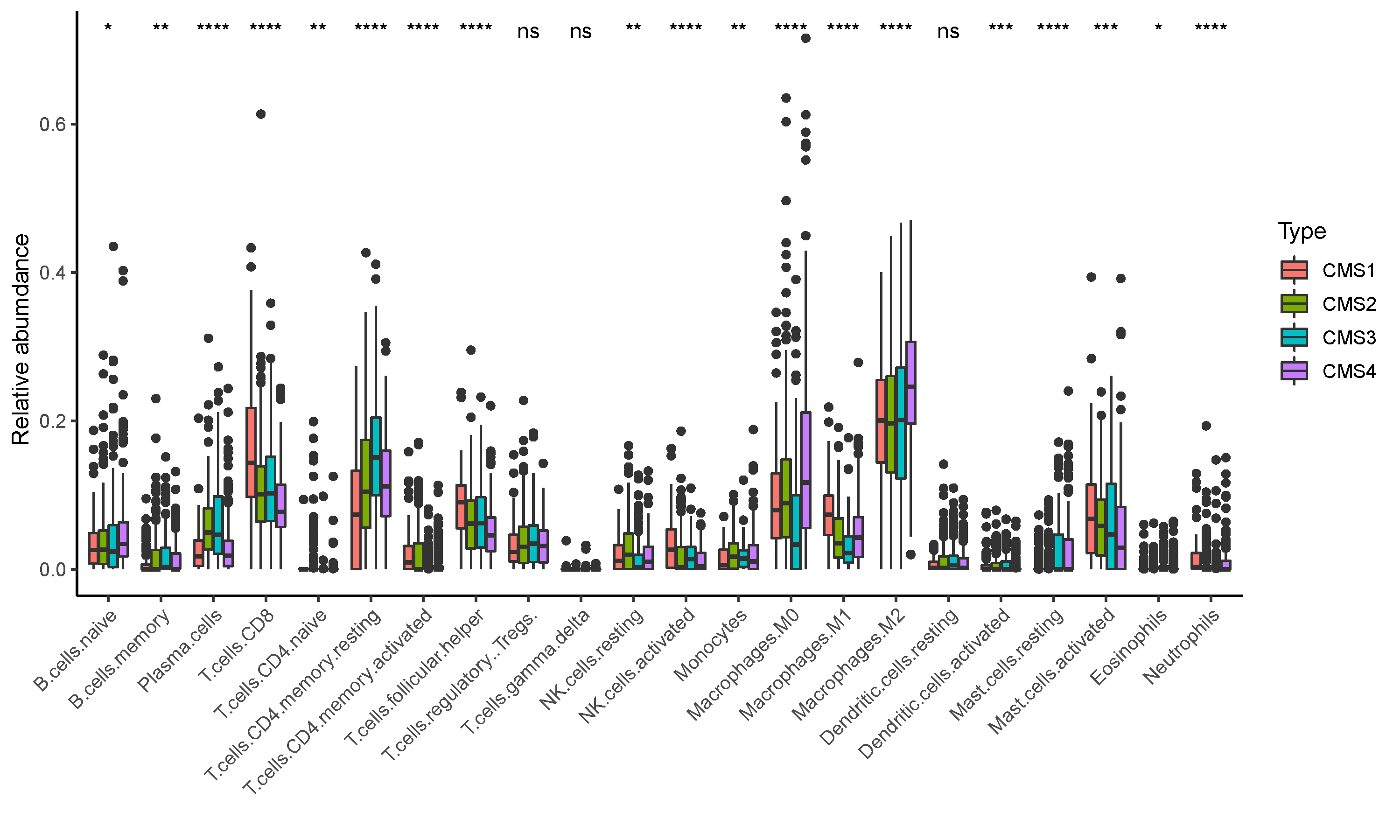

Supplement: Supplementary file 8 [file Image7.TIF]

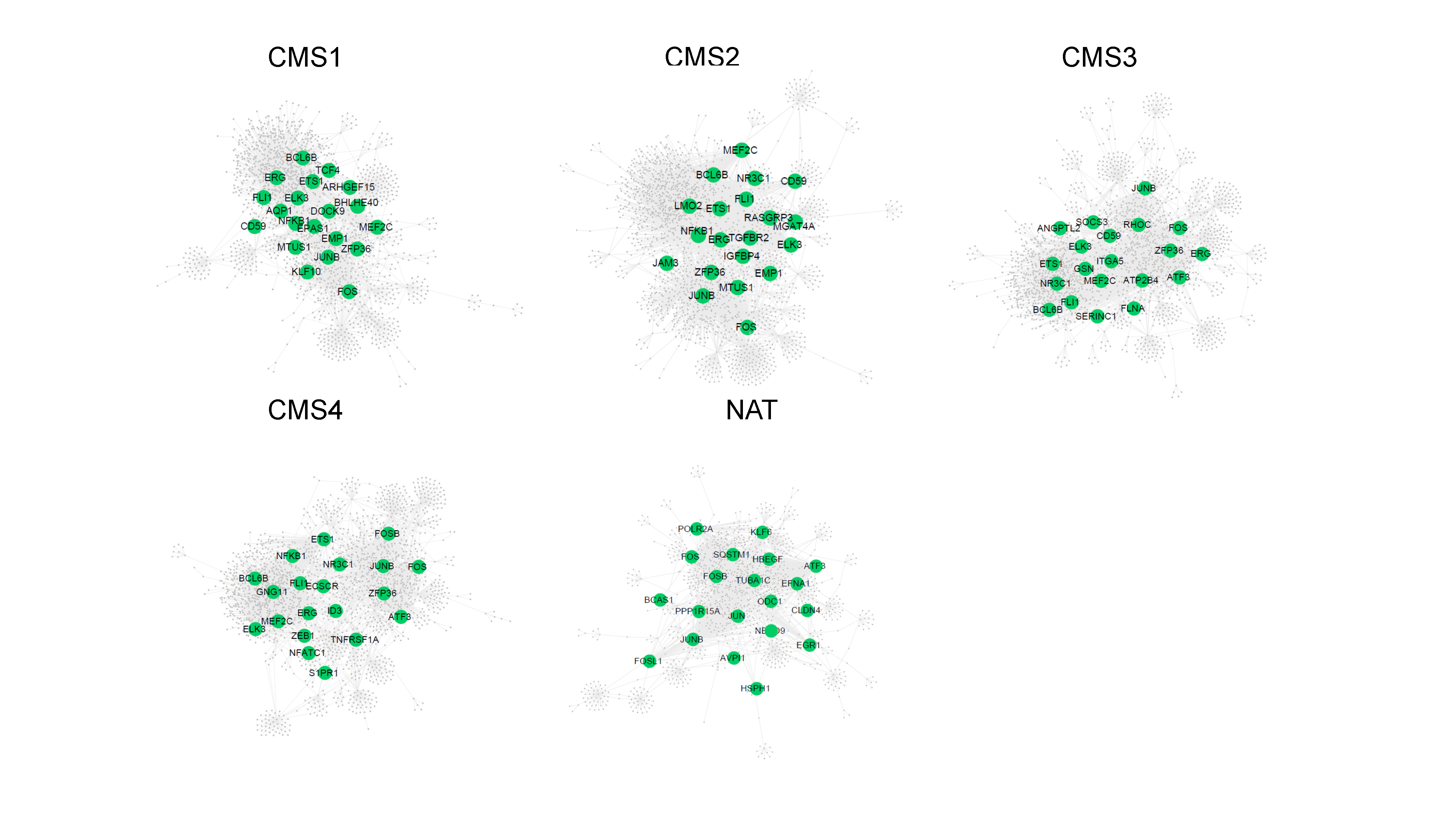

Supplement: Supplementary file 12 [file Image5.TIF]
